# Effect of phthalocyanine, methylene blue and toluidine blue photosensitizers on the adhesive interface of fiber posts: a confocal laser microscopy study

**DOI:** 10.1007/s10103-025-04369-z

**Published:** 2025-02-20

**Authors:** Tuba Gök, Gamze Er Karaoglu, Hulde Korucu

**Affiliations:** 1 Fırat University, Elâzığ, Turkey; 2 Izmir Kâtip Çelebi University, Izmir, Turkey; 3 Sağlık Bilimleri University, Istanbul, Turkey

**Keywords:** Adhesive interface, Confocal laser microscopy, Dentin tubule penetration, Fiber post, Photodynamic therapy, Photosensitizer

## Abstract

Phthalocyanine (Pc) is a promising photosensitizer for photodynamic therapy (PDT), offering advantages such as high light stability, strong absorption, efficient singlet oxygen production, and low dark toxicity. This study evaluated the effects of PDT with different photosensitizers on the penetration depth and area of self-adhesive resin cement into dentinal tubules. Forty single-canal, round-shaped lower premolar teeth were prepared using the ProTaper Next system. After root canals were filled with the single cone technique, samples were stored for 7 days. Post spaces were created, and samples were divided into four groups: control, methylene blue (MB), toluidine blue (TB), and Pc groups. PDT was performed using photosensitizers combined with light-emitting diodes (LEDs). 1.0 mm ± 0.1 axial sections were taken from the 1, 5, and 8 mm levels from the orifice. The penetration depth and area of self-adhesive resin cement into dentinal tubules were analyzed using a confocal laser microscope. Statistical analyses were conducted using ANOVA and Tukey’s test. Cement penetration depth decreased from coronal to apical sections in all groups. At the 1 mm level, MB showed a greater penetration area than Pc, while at the 5 mm level, MB showed a lower penetration area than the control group (*p* < 0.05). No significant differences were found at the 8 mm level. This study showed that while adhesive penetration depth did not differ significantly among groups, penetration area varied at 1 mm and 5 mm sections. Methylene blue exhibited a higher penetration area at 1 mm compared to phthalocyanine, whereas toluidine blue showed consistent penetration across sections. In terms of penetration depth and area findings, the findings showed that PC can be used as an alternative photosensitizer in PDT application in endodontics, showing generally similar findings with MB and TB.

## Introduction

Adequate adhesion of the root canal filling materials to the dentin is essential to prevent the entry of microorganisms and other pathogenic factors into the dentinal tubules [1]. Endodontically treated teeth may require a post-core placement into the root canal depending on the remaining coronal tooth structure [2]. Fiber posts are frequently used due to their elastic modulus similar to the dentin structure, anti-corrosive nature and biocompatibility [3]. As with other root canal filling materials, the adhesion of the fiber post to radicular dentin is critical, and the survival of the post depends on many factors such as presence of smear layer, dentin structure, disinfection methods and resin cement type [4].

It has been reported that sodium hypochlorite (NaOCl) and chlorhexidine digluconate (CHX) solutions used for irrigation of the prosthetic space have negative effects on the bond strength of resin cement to root dentin [5]. Even if irrigation solutions are used in root canals (NaOCl, CHX, ethylene diamine tetra acetic acid (EDTA), etc.), the penetration of the sealer or resin material into the dentinal tubules is limited [6, 7]. Studies have shown that the use of EDTA after NaOCl irrigation removes the smear layer, but subsequently the dentin composition changes, microhardness and flexural strength decrease, and the adhesion between the sealer and dentin is negatively affected [8].

Recent advancements in root canal disinfection include the introduction of new systems and agents such as photodynamic therapy (PDT) [9], which serves as an effective adjunct to routine antimicrobial cleaning for periapical lesion management [10]. Research indicates that photodynamic therapy (PDT) enhances the efficacy of root canal disinfection. This improvement can be attributed to the fact that, in contrast to antibiotics that exert their effects through specific biochemical pathways, the singlet oxygen produced during the photodynamic reaction employs a broad-spectrum physicochemical mechanism. This non-specific mode of action effectively prevents the development of microbial resistance [11, 12]. PDT is based on the principle that light can stimulate a non-toxic dye (photosensitizer) in its target area with minimal photo effect on the surrounding tissue [13]. Photosensitizers is a dye designed to absorb energy from light and transfer this energy to a different molecule in the presence of oxygen [14]. As it reverts to its ground state, the electron facilitates the production of transient, highly reactive oxygen species, termed singlet oxygen. This process results in the irreversible oxidation of targeted cells and microorganisms, significantly impacting their structural integrity [15, 16].

The most investigated dyes in PDT are the phenothiazines (synthetic non porphyrin compounds) methylene blue (MB) and toluidine blue (TB) in various concentrations [17]. In addition to the antimicrobial effects of these photosensitizers, their effects on bond strength or penetration of materials to dentine have also been investigated [18–20]. The studies have indicated that PDT with MB does not affect the ability of self-adhesive cement to penetrate dentin during fiber post cementation [18, 19]. Phthalocyanine (Pc) is a promising photosensitizer for PDT, characterized by its high light stability, strong absorption within the phototherapeutic range (600–900 nm), efficient singlet oxygen production, significant phototoxicity, and minimal dark toxicity [21]. Its antimicrobial efficacy was similar to that of MB and TB, although it was used in a lower concentration [22]. To the best of our knowledge, the effect of phthalocyanine on the penetration ability of resin cement to root canal dentin has not been evaluated in the literature before. Therefore, the aim of this study was to evaluate the effect of different photosensitizers on the adhesive interface between dentin and self-adhesive resin cement. The null hypothesis was that different photosensitizers do not affect the bonding interface of dentin and post cements.

## Materials and methods

### Sample size calculation

The sample size was determined according to a previous study [23]. Considering a type I error (alpha) of 0.05, a power (1-beta) of 0.95, and an effect size of 0.62, a total of 40 in teeth was included for the present study. (WSSPAS; Web-Based Sample Size & Power Analysis Software [24]).

### Sample selection, preparation

The research was approved by the Non-interventional Ethics Committee at Fırat University (process no.2019/06-10) and complied with the Declaration of Helsinki guidelines for studies involving human participants. Fifty single-canaled and round-shaped lower premolar teeth were selected using periapical radiography from the mesial/distal and bucco/lingual aspects. Teeth with root canals in which #10 and #15 K-files got stuck in apical constriction were selected. The study did not include teeth with caries, microcracks on the root surfaces, internal/external root resorption, previous root canal treatment, or immature root apex. The crowns of the teeth were removed with a diamond disc under water cooling (Struers Accutom-3; Struers, Copenhagen, Denmark) and the root lengths were standardized to 15 ± 1 mm.

### Root canal preparation, filling and prosthetic space preparation

A specialist was performed all procedures with at least 5 years of experience in endodontics. The working length was determined with a #10 K-file (Dentsply Maillefer, Ballaigues, Switzerland) was 1 mm shorter than the apical foramen. After providing the glide path with #15 K-file (Dentsply Maillefer), the root canals were prepared using ProTaper Next SX, S1, F1 and F2 rotary file instruments (Dentsply Maillefer), respectively. 2 mL of 1% NaOCl (Werax; Izmir, Turkey) was used between each file change, and 10 mL of 17% EDTA (Werax), 10 mL of 1% NaOCl and 2 mL of distilled water were used as final irrigation. After the root canals were dried with paper points (Diadent; Chongju, Korea), the single cone technique was used to fill the root canals with AH Plus sealer (Dentsply De Trey; Gmbh, Konstanz, Germany) and ProTaper F2 gutta-percha (Dentsply Maillefer). Gutta percha was removed from the orifice level and vertical compaction was achieved with a plugger. Temporary filling was placed in the access cavity and the samples were kept in an environment of 100% humidity, 37 °C for 7 days.

The root canal filling materials were removed using rotary instruments #42.010, and #43.000 (Cytec blanco, Hahnenkratt, Germany) until leaving a remnant of 5 mm in the apical part. The post spaces (10 mm) of the specimens were prepared using post drills 43.001, 43.002 and 43.003 respectively (Cytec blanco), irrigated with 10 mL of distilled water and dried with paper points. The samples were randomly divided into four experimental groups (*n* = 10) (www.randomizer.org).

*Group 1 - (Control group): RelyX U200 Automix*: 43.603 fiber post (Cytec blanco) surface was conditioned with 37% phosphoric acid for 1 min and washed and air dried. Then, silane (RelyX Ceramic Primer; 3 M ESPE, St. Paul, MN, USA) was applied along the post surface using a micro brush for 60 s, and an air jet was gently applied for 5 s. RelyX U200 Automix Self-adhesive resin cement (3 M ESPE) was applied to the post space and the fiber post was placed into the post space.

*Group 2 - PDT (Methylene blue-MB) + RelyX U200 Automix*: The post space was filled with 1mL MB (0.1 mg/mL, 313 µM) MB (Merck KgaA, Darmstadt, Germany) and waited for 5 min. The optical fiber was placed in a static position in the entire post space, and PDT was applied using light-emitting diodes (LED) (Light PDT 630; Easyinsmile International Corp., New Jersey, USA). The LED lamp emitted light with a wavelength range of 620–640 nm (85%), a peak wavelength of 630 nm, and an intensity of 2–4 mW/cm² for 60 s [25]. Methylene blue was aspirated and the post space was irrigated with 5 ml of distilled water for 1 min and dried with a paper point. Post cementation procedure was performed the same as in Group 1.

*Group 3 - PDT (Toluidine blue-TB) + RelyX U200 Automix*: The post space was filled with 1 mL (0.1 mg/mL, 327 µM) TB (Merck KgaA) and waited for 5 min [25]. The PDT application was performed the same as in Group 2 and post cementation procedure was the same as in Group 1.

*Group 4 - PDT (Phthalocyanine-Pc) + RelyX U200 Automix*: The post space was filled with 1 ml of 6 micromolar (µM) Pc (Santa Cruz Biotechnology, Inc., Santa Cruz, CA) and waited for 5 min [26, 27]. The PDT application was performed the same as in Group 2 and post cementation procedure was the same as in Group 1.

In all groups, 0.01% Rhodamine B isothiocyanate pigment was added to RelyX U200 cement before cementation, for later confocal laser microscope examination. Immediately after post-cementation, the roots were centered and placed vertically in a polyvinyl chloride matrix (16.5 diameter x 15.0 mm length) and checked with a parallelogram. The matrices were filled with polyester resin, leaving the cervical 1 mm outside, and the samples were removed from the matrices after 24 h. Axial sections with a thickness of 1.0 mm ± 0.1 were taken from the 1, 5, and 8 mm levels from the orifice using a diamond disc under water cooling.

### Confocal laser analysis

**Fig. 1:**
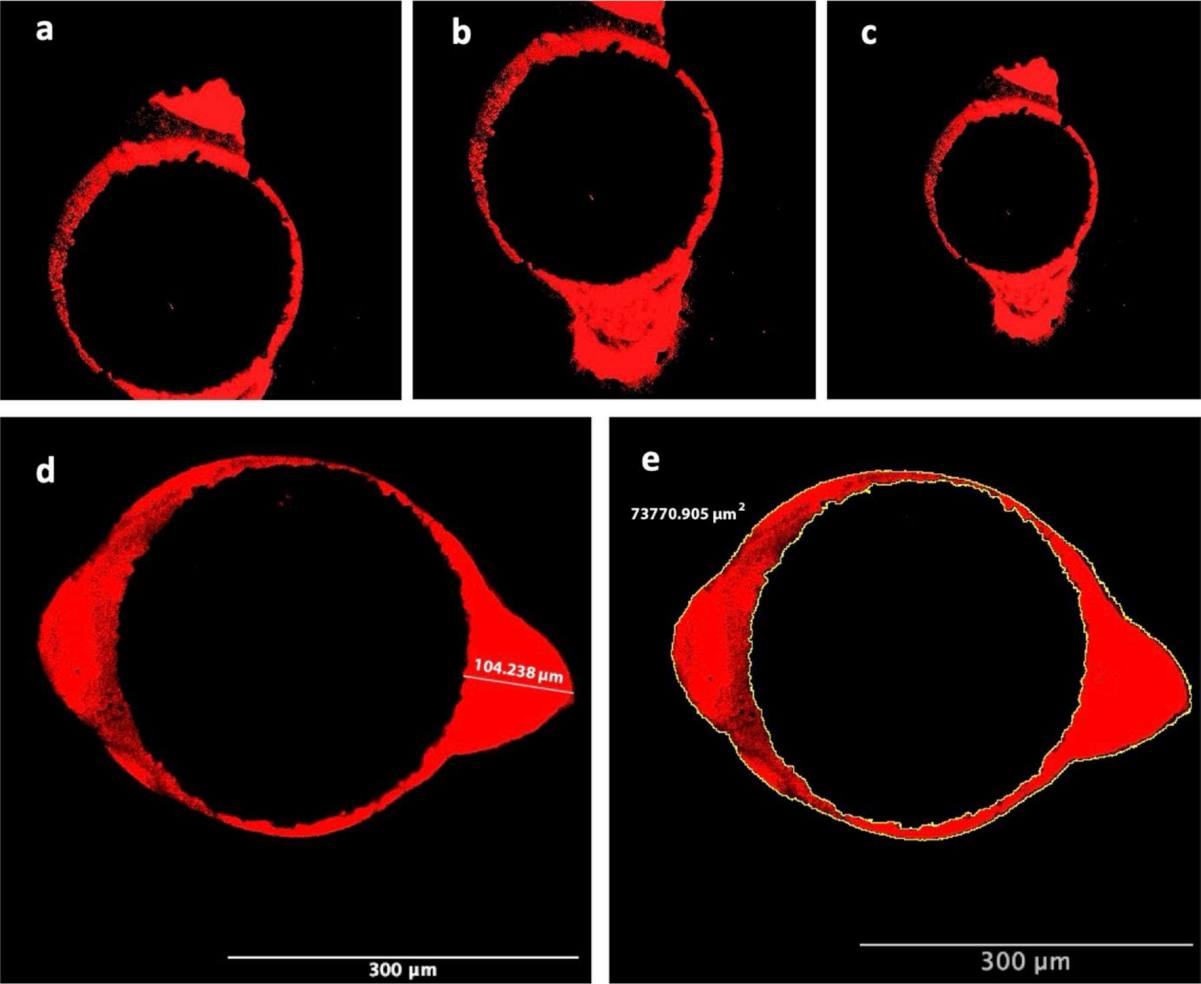
Representative images of creating a single cross-sectional image using Adobe Photoshop software (Adobe Systems Inc) which images could not be displayed in a single image (**a**-**c**). Measurement of maximum dentinal tubule penetration depth (µm) (**d**) and the maximum tubule penetration area (µm^2^) (**e**)

The slices were polished with silicon carbide abrasive paper to produce a smooth surface and rinsed with distilled water. Sections were analyzed using a confocal laser microscope (Carl Zeiss LSM 510; Carl Zeiss Microscopy, Jena, Germany) at 4X magnification with an Argon laser light source emitting at a wavelength range of 560–600 nm to determine sealer penetration around the root canal and within the dentinal tubules. For sections where the entire root canal could not be visualized within a single image, multiple images were merged into a composite photograph using Adobe Photoshop software (Adobe Systems Inc., San Jose, CA, USA), resulting in a single section image (Fig. 1a-c). The maximum tubule penetration depth (µm) (Fig. 1d) and the maximum tubule penetration area (µm^2^) (Fig. 1e) were quantified using Image J software (National Institutes of Health, Bethesda, MD) [7]. The CLSM analysis was performed by one examiner who was blinded to the groups.

### Data presentation and statistical analysis

The data were normally distributed (Shapiro-Wilk, *p* > 0.05) and showed homogeneity of variance (Levene’s test, *p* > 0.05), parametric tests were applied. The ANOVA one-way test was used to evaluate the penetration depth, and area of the adhesive interface of different photosensitizers. The post hoc Tukey test was used for multiple comparisons. Statistical tests were performed using SPSS for Windows, Version 26.0 (IBM Corp., Armonk, NY, USA), and the cut-off level for significance was set at α = 5%.

## Results

The mean and standard deviation of dentinal tubule penetration depths, and areas at 1, 5 and 8 mm in the different groups are shown in Table 1.

**Table 1:** Mean and standard deviation (SD) of the penetration depth and area of different photosensitizers at the adhesive interface between dentin and self-adhesive resin cement (*n* = 10)

	**Penetration depth (mm)**			**Penetration area (mm** ^**2**^ **)**		
	1 mm	5 mm	8 mm	1 mm	5 mm	8 mm
	Mean ± (SD)			Mean ± (SD)		
**Control**	0.45 ± 0.09^a^	0.10 ± 0.01^a^	0.04 ± 0.01^a^	0.73 ± 0.10^ab^	0.08 ± 0.01^a^	0.02 ± 0.00^a^
**MB**	0.49 ± 0.13^a^	0.10 ± 0.02^a^	0.04 ± 0.01^a^	0.85 ± 0.13^a^	0.06 ± 0.01^b^	0.02 ± 0.00^a^
**TB**	0.50 ± 0.08^a^	0.12 ± 0.03^a^	0.05 ± 0.01^a^	0.79 ± 0.12^ab^	0.07 ± 0.01^ab^	0.02 ± 0.00^a^
**Pc**	0.37 ± 0.07^a^	0.12 ± 0.03^a^	0.04 ± 0.01^a^	0.66 ± 0.11^b^	0.07 ± 0.01^ab^	0.02 ± 0.00^a^
*P* value	0 0.014	0 0.298	0.010	0.002*	< 0.001*	0 0.695

Different letters indicate the significant differences among the gap areas in each column (*p* < 0.05). Abbreviations: MB, methylene blue; TB, toluidine blue; Pc, phthalocyanine. *Significant at *p* < 0.05

### Evaluation of dentinal tubule penetration

**Fig. 2:**
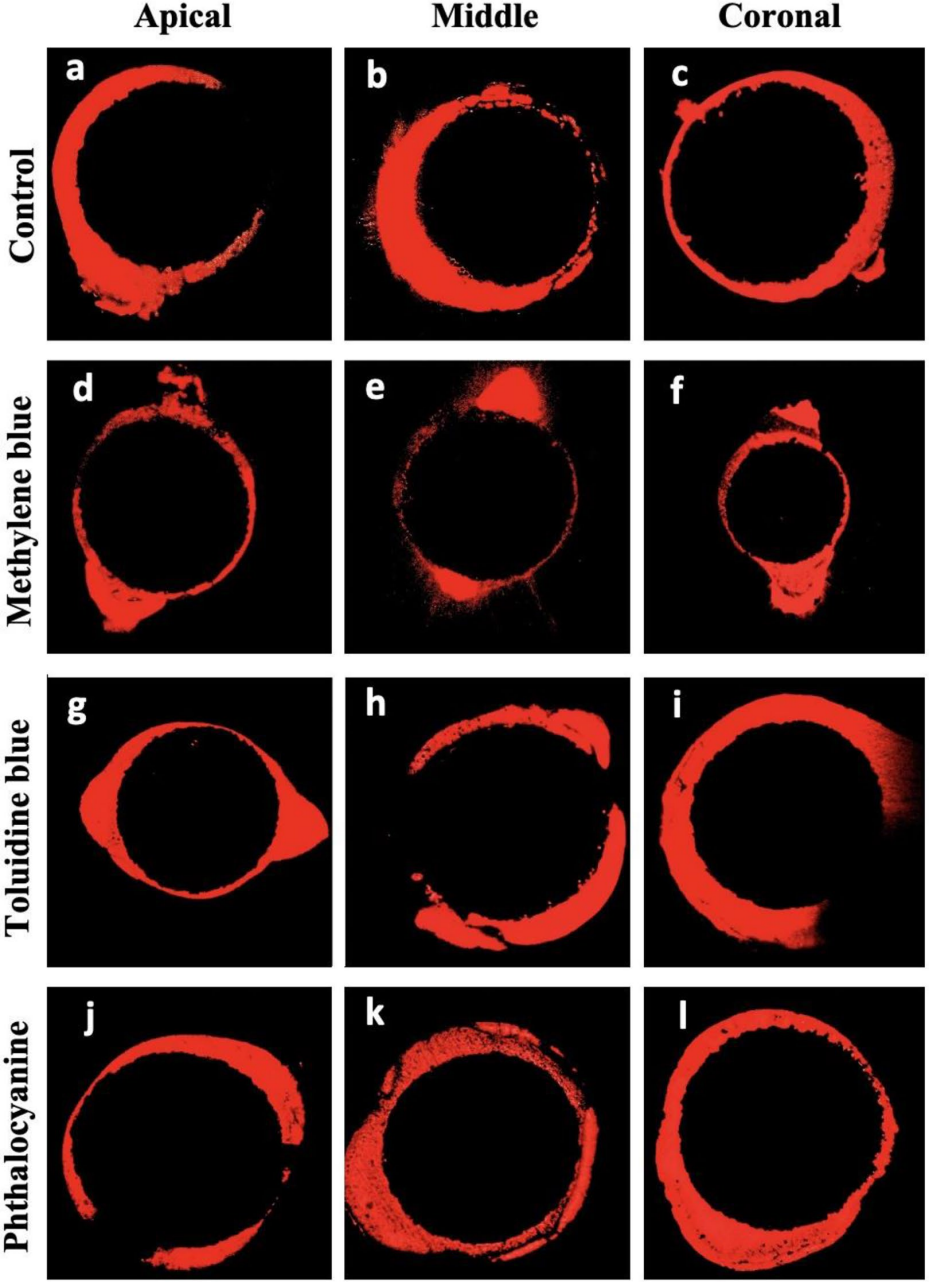
Representative confocal laser scanning microscopy images of each experimental group at apical, middle, and coronal regions

There was no statistically significant difference in adhesive cement penetration depth at 1, 5 and 8 mm sections among groups (*p* > 0.05). However, within each group, a progressive decrease in penetration depth was observed from coronal to apical Sections  (8 mm < 5 mm < 1 mm), indicating that the adhesive cement penetrated the dentinal tubules more effectively in the coronal region compared to the apical region (Fig. 2).

At 1 mm sections, the penetration depth was slightly higher in the MB and TB groups compared to the control and Pc groups, although the difference was not statistically significant (*p* > 0.05). The TB and Pc groups exhibited greater penetration depth than the other groups at 5 mm sections, yet no significant differences were noted (*p* > 0.05). At 8 mm, all groups demonstrated the lowest penetration depth values, with no statistical differences among them (*p* > 0.05).

### Evaluation of penetration area

While there was no significant difference in the penetration area of the adhesive cement at 8 mm sections (*p* > 0.05), there were significant differences among the groups at 1 and 5 mm sections (*p* < 0.05). MB group showed higher penetration area than the Pc groups at 1 mm sections (*p* < 0.05). At 5 mm sections, the MB group demonstrated a smaller penetration area compared to the control group (*p* < 0.05), suggesting that in the middle third, the control group achieved a more extensive distribution of adhesive cement. However, no significant differences were detected between the MB, TB, and Pc groups (*p* > 0.05).

The penetration area decreased from coronal to apical sections in all groups (8 mm < 5 mm < 1 mm) (Fig. This pattern suggested that the diffusion capacity of adhesive cement diminished as it advances towards the apical portion of the dentinal tubules.

## Discussion

Antimicrobial PDT involves the absorption of photons by a specific photosensitizer when exposed to light of a particular wavelength. This photon absorption excites electrons within the photosensitizer, which transfers energy to the dental surface [28]. In environments rich in oxygen, this energy transfer initiates the formation of reactive oxygen species, including singlet oxygen. These highly reactive molecules cause irreversible oxidation, damaging microbial cells and effectively reducing the overall microbial population [20]. Furthermore, reactive oxygen species play a key role in facilitating intermolecular cross-linking among adjacent collagen fibrils, thereby enhancing collagen’s resistance to enzymatic breakdown. Since collagen fibrils are fundamental structural proteins in the dentin matrix, this increased cross-linking may stabilize the shear bond strength and mechanical properties of endodontic materials within intraradicular dentin treated with PDT [29]. Therefore, PDT can be utilized as an alternative method for root canal decontamination and post space disinfection.

Studies have indicated that utilizing PDT as an additional disinfection step in endodontic treatment is an effective strategy for enhancing root canal disinfection [30, 31]. To our knowledge, no studies have evaluated the penetration of post cement adhesive into dentinal tubules following post space disinfection using PDT with MB, TB or Pc. Therefore, this study investigated the effects of PDT application using different photosensitizing agents in the post space on the penetration depth and area of self-adhesive resin cement into dentinal tubules. While no differences were found between the groups in the assessment of penetration depth, statistically significant differences were observed among the groups in terms of penetration area. Therefore, the null hypothesis was partially rejected.

Within PDT, the predominant and most extensively investigated photosensitizers are phenothiazines—specifically the synthetic non-porphyrin agents MB and TB administered at multiple concentrations [17]. Although their hydrophilic nature and ability to enhance antimicrobial efficacy as an adjunct to routine endodontic treatment are well recognized, the search for alternative photosensitizers continues due to their potential to cause tooth discoloration [32]. In this study, Pc is employed as an alternative photosensitizer due to its strong absorption capacity in the phototherapeutic window, high light stability, high phototoxicity, low dark toxicity and singlet oxygen-producing efficiency [21, 33].

In this study, MB and TB were applied at a 0.1 mg/mL concentration. A previous study have confirmed that these concentrations are sufficient to achieve significant antimicrobial efficacy [22]. Considering the potential for these photosensitizers to cause tooth discoloration [32], their application at low concentrations in this study was considered appropriate to mimic in vivo dosage conditions. A review of the literature reveals that MB and TB have been used at highly variable concentrations in studies with similar objectives [34, 35]. This highlights the need for further in vitro and in vivo research to establish standardized protocols.

In our comprehensive literature review, there is no study comparing MB, TB, and Pc with PDT. Therefore, the results of our study cannot be directly compared with those of other studies in the literature. As a result of our study, we found that there was no statistically significant difference in adhesive cement penetration depth at 1, 5 and 8 mm sections among groups. In each group, the penetration depth and penetration area of adhesive cement decreased from the coronal to the apical direction. Consistent with this study, Al Ahdal et al. [36] found that the extrusion bond strength decreases from the coronal to the apical region. Similarly, Alonaizan et al. [37] also indicated that in the cervical segments, the mean push-out bond strength was slightly higher for experimental groups compared to apical segments. A credible explanation for this outcome may be the more effective removal of photosensitizers from the coronal region compared to the apical region. Additionally, the lower quantity and higher density of dentinal tubules in the apical region may also contribute to this outcome.

In the current study, the maximum penetration area of adhesive cement in the coronal section was higher in the MB group compared to the Pc group. Considering the adhesive resin cement bonding, the hydrophilic nature of MB [38] may mimic the effects of primer application, which could explain the better bonding results observed in the coronal region in this group compared to the Pc group. On the other hand, Pc, due to its lower hydrophilicity may negatively affect resin bonding. Contrary to these findings, Almadi et al. reported that the MB group exhibited lower extrusion bond strength compared to the TB, curcumin, and phycocyanin groups [39]. Similarly, Ahdal et al. [36] stated that the MB group exhibited lower bond strength compared to the TB and the curcumin groups. The probable explanation for this discrepancy is the use of photosensitizer at different concentrations in the studies. The hydrophilic nature of MB [38] negatively affects the bonding of adhesive resin cement. This property of MB varies proportionally with the concentration used [40]. Therefore, it can be suggested that MB, when used as a photosensitizer at higher concentrations, may lead to lower bond strength. In the studies under discussion, the concentrations of MB were 100 mg/L [39] and 50 mg/L [36], respectively. In our study, however, MB was used at a concentration of 0.1 mg/ml, which may have influenced its effect on bond strength.

This study has certain inherent limitations due to its in vitro experimental design. Thus, additional in vivo and ex vivo studies conducted under conditions that mimic the oral environment are suggested to corroborate the results of the current study. Evaluating the physical effects of various photosensitizers on radicular dentin, in conjunction with mechanical assessments such as fracture resistance, elastic modulus, and microhardness, may offer significant insights into the comprehensive impact of these photosensitizers. Additionally, the bond strength of different root canal sealers to dentin, when combined with various photosensitizers, can be a topic for future investigation. Furthermore, different light sources and photosensitizers used at varying concentrations may also influence bond strength. Therefore, in vitro and in vivo studies are necessary in this regard. The strength of this study lies in examining the effect of Pc, known for its antimicrobial efficacy [22], on bond strength and investigating whether it could serve as an alternative to photosensitizers that, despite causing discoloration, continue to be widely used.

## Conclusion

Pc demonstrated comparable effects to MB and TB in terms of dentinal tubule penetration area, with no significant differences observed in penetration depth.

## Data Availability

The data supporting this study’s findings are available from the corresponding author upon reasonable request.
